# Prenatal Exposure to Valproic Acid may Alter CD200/CD200R Signaling Pathways in a Rat Model of Autism Spectrum Disorder

**DOI:** 10.31083/AP39444

**Published:** 2025-04-01

**Authors:** Xiaoou Xu, Li Tan, Xiaojuan Zhang

**Affiliations:** ^1^Department for Science and Technology Management and Education, Chongqing Population and Family Planning Science and Technology Research Institute, 401120 Chongqing, China; ^2^Department of Clinical Medicine, Chongqing Medical and Pharmaceutical College, 401331 Chongqing, China

**Keywords:** autism spectrum disorder, valproic acid, microglia, neuron, CD200, CD200R, CX3CL1, CX3CR1, neuroinflammation

## Abstract

**Objective:**

To investigate the potential toxic effects of prenatal exposure to valproic acid (VPA) on microglia-neuron communication in the brain, with a specific focus on the alterations in key molecules involved in this process, namely CX3CL1/CX3CR1 and CD200/CD200R, during the early stages of life in a rat model of autism.

**Methods:**

Pregnant female rats were administered either sterile saline or VPA on embryonic day 12.5. The brains of the rat offspring were collected on postnatal day 30 for analysis. Immunohistochemical techniques and enzyme-linked immunosorbent assay (ELISA) were employed to assess changes in microglia-neuron crosstalk.

**Results:**

The study revealed a significant reduction in CD200 levels within the hippocampus of rats on postnatal day 30 following prenatal exposure to VPA, indicating an impairment in CD200/CD200R signaling. Additionally, there was no observed increase in microglial numbers or any pathological alterations in the hippocampus. Additionally, no significant changes in the levels of CX3CL1 and CX3CR1 were noted in the VPA-exposed rats compared with the control group.

**Conclusion:**

Prenatal exposure to VPA resulted in a decrease in CD200 expression within the hippocampus, potentially disrupting the communication between microglia and neurons. The findings suggest that VPA may modify the interactions between microglia and neurons, which could lead to neuroinflammation due to hyperactivated microglia. These disruptions have the potential to affect synaptic connectivity and contribute to the development of neurodevelopmental disorders, including autism. Further research is necessary to clarify the underlying mechanisms and implications for pathological conditions associated with autism spectrum disorder (ASD).

## Main Points

1. CD200/CD200R signaling is altered in autism spectrum disorder (ASD), potentially disrupting the communication between microglia and neurons.

2. The disruption of CD200/CD200R signaling may play an important role in the development of ASD.

3. CX3CL1/CX3CR1 signaling was not involved in the disruption of the intercommunication between microglia and neurons in a rat model of autism induced by valproic acid (VPA).

## 1. Introduction

Autism spectrum disorder (ASD) represents a complex and varied set of neurodevelopmental conditions typified by deficiencies in both verbal and nonverbal communication and social interaction impairments, as well as the presence of stereotyped repetitive behaviors and restricted areas of interest [1, 2]. The precise etiology of ASD remains poorly understood; nevertheless, genetic, environmental, and immunological factors have been implicated as potential contributors. To date, there is no established effective treatment for ASD. Given the chronic nature of ASD from early in life, studies have predominantly centered on childhood to investigate the etiology, pathophysiology, and therapeutic approaches for ASD in both human subjects and animal models. Valproic acid (VPA) is a well-known pharmaceutical agent commonly used for the management of epilepsy and various seizure disorders. However, exposure to VPA during embryonic development has been shown to induce autism-like symptoms in animal models. The VPA model of autism is one of the most extensively employed animal models in this area of research. Offspring exposed to VPA display significant behavioral changes closely resembling core symptoms of ASD, including pronounced social impairments and stereotyped behaviors [3].

Within the realm of investigating the pathophysiological basis of ASD, a growing body of research has implicated neuroinflammation as a significant factor, operating through various neurobiological mechanisms [4, 5]. Numerous studies have indicated that brain inflammation can adversely affect synaptic function, potentially leading to cognitive impairments and behavioral abnormalities [6, 7, 8]. The immune response of the central nervous system (CNS) is mediated by microglial cells, which serve as the primary line of defense against pathological alterations [9, 10]. Elevated levels of activated microglia have been reported in postmortem tissue from individuals with ASD, as well as in various animal models. Within the CNS, key signaling molecules involved in neuron-microglia communication, such as CX3CL1/CX3CR1 and CD200/CD200R, play a vital role in suppressing microglial activation and mitigating neuroinflammation [11, 12, 13]. CX3CL1 (fractalkine) demonstrates significantly higher expression in the brain compared with peripheral tissues, suggesting a distinct role for this ligand in the CNS. CX3CL1 is primarily derived from neurons, while its sole known receptor, CX3CR1, is found on microglial cells. CD200 is a membrane glycoprotein expressed widely on neurons. The corresponding receptor of CD200 (CD200R) is predominantly found in microglia. Impairment of the CD200-CD200R and CX3CL1-CX3CR1 signaling pathways has been implicated in inducing excessive reactivity of microglial cells [14]. Despite the growing body of research focused on elucidating the pathological alterations affecting microglia and neurons in ASD, including neuronal proliferation, migration, differentiation, and the establishment and maturation of synaptic networks, investigation into the role of CX3CL1/CX3CR1 and CD200/CD200R signaling pathways, which are involved in maintaining proper microglial reactivity levels in the VPA-induced ASD model, has yet to be conducted.

The main aim of this study is to explore the potential toxic effects of prenatal exposure to VPA on the intercommunication between microglia and neurons in the brain, specifically focusing on the alterations in key molecules involved in this process, namely CX3CL1/CX3CR1 and CD200/CD200R, during the early stages of life in a rat model of autism. We hypothesize that prenatal exposure to VPA will elicit changes in the expression and function of CX3CL1/CX3CR1 and CD200/CD200R in this particular model. Our findings reveal a significant reduction in CD200 levels within the hippocampus of rats on postnatal day 30, following prenatal exposure to VPA. This reduction indicates an impairment in CD200/CD200R signaling within the hippocampus of the autistic rat model. Concurrently, we did not observe an increase in the number of microglia, which aligns with previous studies. Collectively, these results suggest that environmental agents, such as VPA, can induce alterations in the intercommunication between microglia and neurons, with potential consequences including neuroinflammation caused by hyperactivated microglia. These disruptions have the potential to profoundly impact neurons and synaptic connectivity, thereby contributing to the development of neurodevelopmental disorders, including autism.

## 2. Materials and Methods

### 2.1 Animals

Pairs of adult male and female rats were housed for breeding. Female breeders underwent daily visual examination prior to 8:00 a.m. to ascertain the presence of a vaginal plug, which was documented as embryonic day 0 (E0). Pregnant females were administered either sterile saline or VPA (500 mg/kg) dissolved in sterile saline, via subcutaneous injection on E12.5 at a volume of 10 mL/kg, according to a previously established protocol [5]. Each cage contained 2–3 pregnant rats, with each pregnant rat giving birth to 10–16 offspring. Offspring within each group were from the same pregnant rat. The day of birth was designated as day 0 (P0) in accordance with previous research [5]. All animals were accommodated at South West University in environmentally controlled rooms, maintaining a 12-hour light/dark cycle, regulated temperature, and humidity. Water and laboratory chow were provided ad libitum. The experimental protocols involving the rats were approved by the institutional animal care and use committee of Southwest University in China (No: IACUC-20221010-06). Every effort was made to minimize animal suffering, limit the number of animals used, and explore alternative techniques to *in vivo* methods whenever possible.

### 2.2 Tissue Collection

Offspring rats were selected randomly. Three female rats and three male rats were chosen for the control group. There were three female rats and three male rats in the VPA group. The brains of rat offspring were collected on postnatal day 30 (P30) (n = 6 brains/group; number of sections/brains used = 6). During the tissue collection procedure, the rats were placed under anesthesia (0.1 mL/10 g, 10% chloral hydrate, Macklin, Shanghai, China). The rats were perfused transcardially with cold 0.9% saline followed by 4% paraformaldehyde. The animals were decapitated, and their brains were extracted and immersed in cold 4% paraformaldehyde for 4 hours. Subsequently, the brains were moved to a refrigerator at –80 °C.

### 2.3 Social Ability Test 

We assessed social approach using our automated three-chambered apparatus on postnatal day 29 (P29), following methods outlined in previous studies [15]. Entry counts and time spent in each chamber were recorded automatically, using photocells integrated into the doorways, and analyzed by trained personnel. Testing began with a 10-minute habituation session in the central chamber, followed by a similar session in all three empty chambers. Subsequently, the subject was temporarily confined to the central chamber while a clean novel object was placed in one side chamber, and a novel rat, aged 4–6 weeks and previously acclimated to the environment, was placed in an identical wire cup in the other side chamber. After positioning both stimuli, the side doors were lifted simultaneously, granting the subject access to all three chambers for 10 minutes. Time spent and entries into each chamber were automatically recorded. Furthermore, the duration of sniffing behavior directed towards the novel object and novel rat during the 10-minute test session was later scored by a trained observer using video recordings.

### 2.4 Immuno-Histochemical Detection of Protein

The brains were sectioned at –20 °C with a thickness of 20 µm. All sections were stained under identical conditions. To stain the slides, they were thawed at room temperature for 10 minutes, washed with PBS, and then incubated for 1 hour in PBS with 5% normal goat serum. After blocking, the slides were incubated overnight at 4 °C with a primary antibody (rabbit anti-IBA1 at a 1:100 dilution, Biotechnology Company, Beijing, China). On the following day, the slides were washed and incubated with a goat anti-rabbit secondary antibody for 30 minutes. Diaminobenzidine (DAB) drops were applied to the tissue, resulting in a brownish-yellow coloration. The cell nucleus was stained with hematoxylin for 3 minutes.

The sections were photographed using the Motic Group’s microcamera system (BA400Digital, MOTIC CHINA GROUP CO., LTD., Xiamen, Fujian, China). A total of three images were obtained by first observing each section at ×100 magnification and then capturing images at both ×100 and ×400 magnification. It was determined what proportion of the tissue had positive DAB staining. The Indica Labs HALO® (version 3.0.311.407, Albuquerque, NM, USA) was used to determine the percentage of positive area (DAB Positive Tissue%) in each ×400 magnification image. The nuclei stained with hematoxylin appeared blue, while areas of positive DAB expression appeared brown.

### 2.5 Enzyme-Linked Immunosorbent Assay (ELISA)

IBA1, CD200, CD200R, CX3CL1, and CX3CR1 were quantified using commercial ELISA kits (Fine Biotech, Wuhan, Hubei, China) following the manufacturer’s instructions.

### 2.6 Histopathological Analysis

After dehydration and embedding in paraffin, the harvested brain tissues were fixed with 10% neutral-buffered formalin. Then, for histological examination, the embedded brain slices (5-µm thickness) were stained with hematoxylin and eosin (HE). The hippocampal CA1, CA2, and CA3 regions were then analyzed.

### 2.7 Statistical Analysis

Statistical analyses were performed using SPSS 20.0 software for Windows (SPSS, Inc., Chicago, IL, USA). In this study, continuous data were presented as median [Q1–Q3]. The Mann-Whitney U test was used to analyze the differences between groups, and the significance level was set at *p* < 0.05.

## 3. Results

### 3.1 Prenatal VPA Exposure Impairs Social Abilities in Offspring Rats

**Fig. 1.**
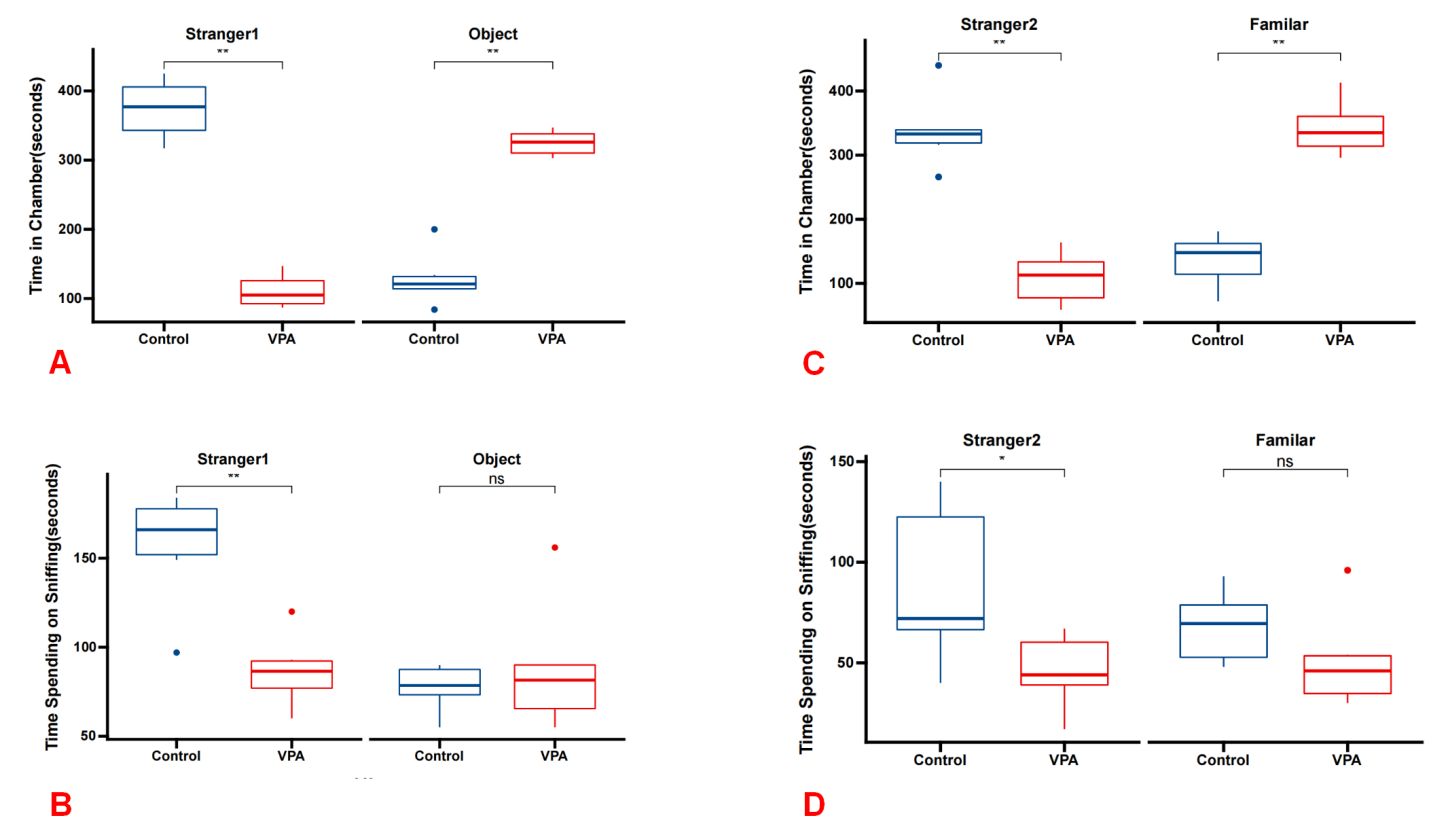
**Social deficits in the VPA group rats demonstrated across the three-chamber social test paradigm**. (A) In Phase 1, VPA group rats exhibited significantly reduced interaction time with stranger 1 compared with the control group, while showing increased interaction time with the object (n = 6 for each group). (B) During Phase 1, VPA group rats spent significantly less time sniffing stranger 1 compared with the control group, while spending more time sniffing the object. (C) In phase 2, VPA group rats displayed significantly reduced interaction time with stranger 2 compared with the control group, while exhibiting increased interaction time with the familiar object. (D) During phase 2, VPA group rats spent significantly less time sniffing stranger 2 compared with the control group, while spending more time sniffing the familiar object. *p < 0.05 vs control group, **p < 0.01 vs control group. ns, no significance; VPA, valproic acid.

The three-chamber social experiment revealed significant social deficits in the VPA group of rats compared with the control group. During Phase 1, the VPA group rats exhibited reduced interaction time with stranger 1 (median [Q1–Q3]: 377 [329.75, 416] seconds) compared with the control group (median [Q1–Q3]: 105 [88.5, 135.75] seconds; *p* = 0.005). In contrast, they showed increased interaction time with the object (median [Q1–Q3]: 121 [105.75, 150.5] seconds) compared with the control group (median [Q1–Q3]: 326 [306, 341.75] seconds; *p* = 0.005). Furthermore, the VPA group spent less time sniffing stranger 1 (median [Q1–Q3]: 166 [136, 181] seconds) compared with the control group (median [Q1–Q3]: 16.5 [71.25, 99.75] seconds; *p* = 0.0031). In Phase 2, rats in the VPA group exhibited a significantly reduced interaction time with stranger 2 (median [Q1–Q3]: 333 [303.5, 365] seconds) compared with the control group (median [Q1–Q3]: 113 [65, 145.25] seconds; *p* = 0.005). Additionally, they demonstrated an increased interaction time with a familiar rat (median [Q1–Q3]: 148 [95.25, 170.5] seconds) compared with the control group (median [Q1–Q3]: 335 [308, 376.25] seconds; *p* = 0.005). Consistently, the VPA group spent less time sniffing stranger 2 (median [Q1–Q3]: 118 [61, 152.5] seconds) compared with the control group (median [Q1–Q3]: 35 [6.75, 64.75] seconds; *p* = 0.045). These findings suggest that prenatal exposure to VPA induces social deficits in rats, as evidenced by altered social interaction patterns and sniffing behaviors observed in the three-chamber social experiment (Fig. 1).

### 3.2 The Administration of VPA did not Result in a Reduction in the Number of Microglia in the Hippocampus of Rats

**Fig. 2.**
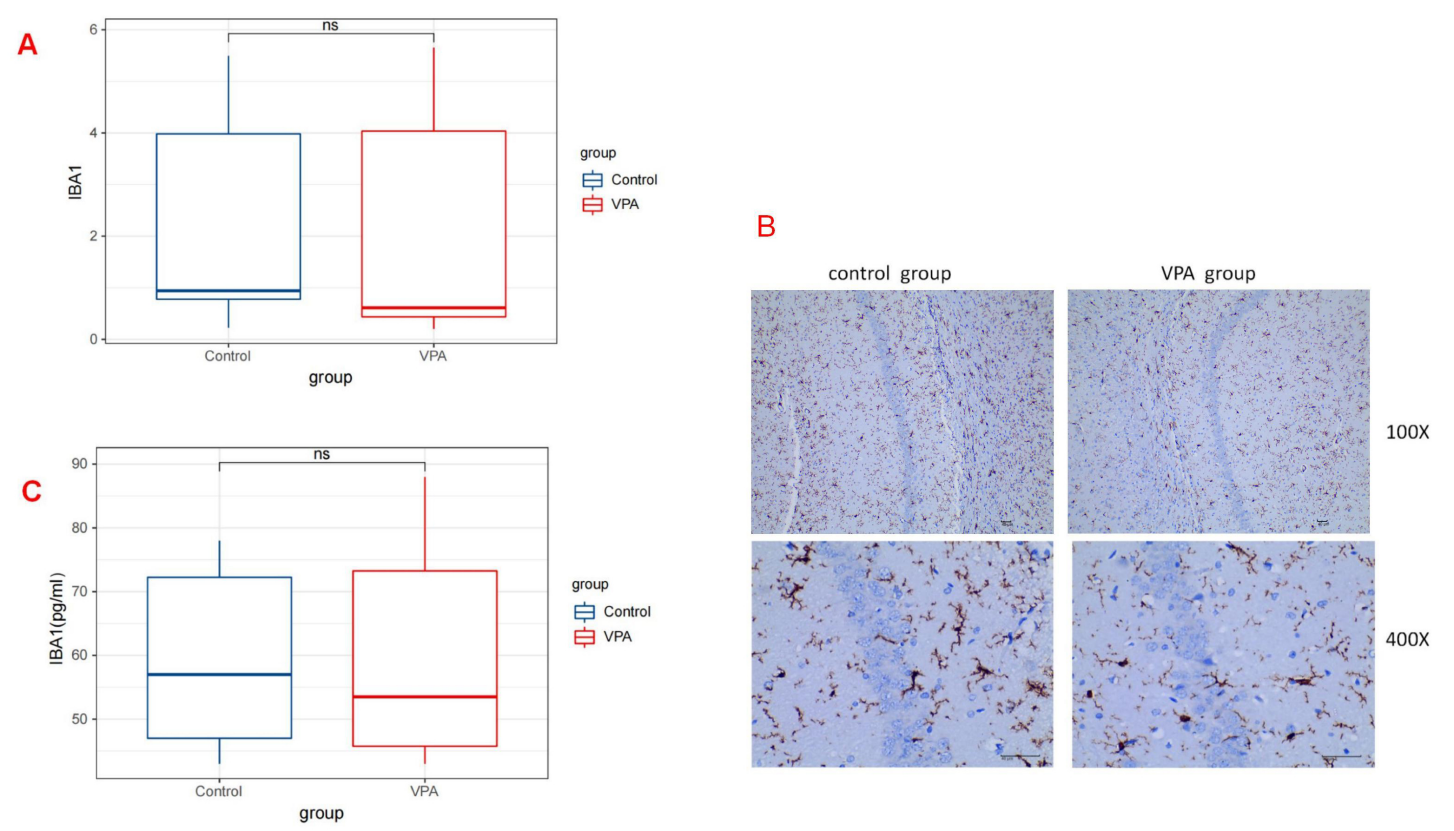
**IBA1 expressions in the Rat Hippocampus**. (A) Illustration of the quantitative immunohistochemical analysis of IBA1, representing the number of microglia in the hippocampus on postnatal day 30 (P30). No statistically significant difference was observed in the number of microglial cells between rats treated with VPA and those treated with saline (*p* > 0.05). (B) Depiction of the immunohistochemical photomicrographs of microglia from both the VPA group and the control group. Scale bar: 40μm. (C) Levels of IBA1 by ELISA between the VPA group and the control group (*p* > 0.05). There was no statistically significant difference in the expression level of IBA in the hippocampus between the two groups, indicating that exposure to VPA during pregnancy had no significant effect on the number of microglia in the hippocampus of rats offspring. ns, no significance.

The hippocampus is widely recognized as the primary region responsible for learning and memory. Studies have identified impaired hippocampal function in both autistic children and in a rat model of autism induced by VPA [13]. The quantitative results of immunohistochemistry indicated the expression levels of IBA1 on postnatal day 30 in rats treated with VPA (median [Q1–Q3]: 0.94 [0.60–5.12]) did not exhibit a statistically significant difference compared with those in rats treated with saline (median [Q1–Q3]: 0.61 [0.35–5.27]; *p* = 0.589) (Fig. 2A) (Table 1). ELISA results also demonstrated no significant difference between the two groups (median [Q1–Q3]: 48.00 [43.75, 59.25] in the control group vs 57.00 [46.75, 80.50] in the VPA group; *p* = 0.893) (Fig. 2C) (Table 2). The photomicrographs depicting IBA1-positive microglia (stained in brownish-yellow) in the hippocampus of rats treated with saline and VPA on postnatal day 30 are shown in Fig. 2B.

**Table 1.** **Levels of IBA1, CD200, CD220R, CX3CL1, and CX3CR1 by immunohistochemistry**.

	Control group	VPA group	*p*
	(n = 6)	(n = 6)	
IBA1	0.94 (0.60–5.12)	0.61 (0.35–5.27)	0.441
CD200	11.27 (7.62–13.29)	6.64 (5.40–7.51)	0.031
CD200R	3.99 (2.69–10.92)	2.14 (1.11–10.89)	0.441
CX3CL1	13.19 (9.67–21.95)	14.75 (10.86–23.70)	0.893
CX3CR1	4.77 (2.29–15.24)	3.39 (2.22–16.47)	0.893

**Table 2.** **Levels of IBA1, CD200, CD220R, CX3CL1, and CX3CR1 by ELISA**.

	Control group	VPA group	*p*
	(n = 6)	(n = 6)	
IBA1	48.00 (43.75–59.25)	57.00 (46.75–80.50)	0.893
CD200	152.50 (137.25–181.75)	101.50 (94.00–111.25)	0.031
CD200R	156.00 (123.00–205.75)	100.00 (86.25–132.75)	0.139
CX3CL1	138.47 (117.46–174.84)	122.89 (63.75–146.20)	0.441
CX3CR1	93.83 (82.31–100.90)	73.38 (59.46–118.44)	0.139

ELISA, enzyme-linked immunosorbent assay.

### 3.3 Valproic Acid Treatment Impairs CD200/CD200R Signaling in the Rat Hippocampus

The interaction between CD200 and CD200R serves as a vital inhibitory signal that helps to keep microglia in a resting state. A decrease in the levels CD200 or CD200R can lead to the abnormal activation of microglia, subsequently triggering a neuroinflammatory response.

**Fig. 3.**
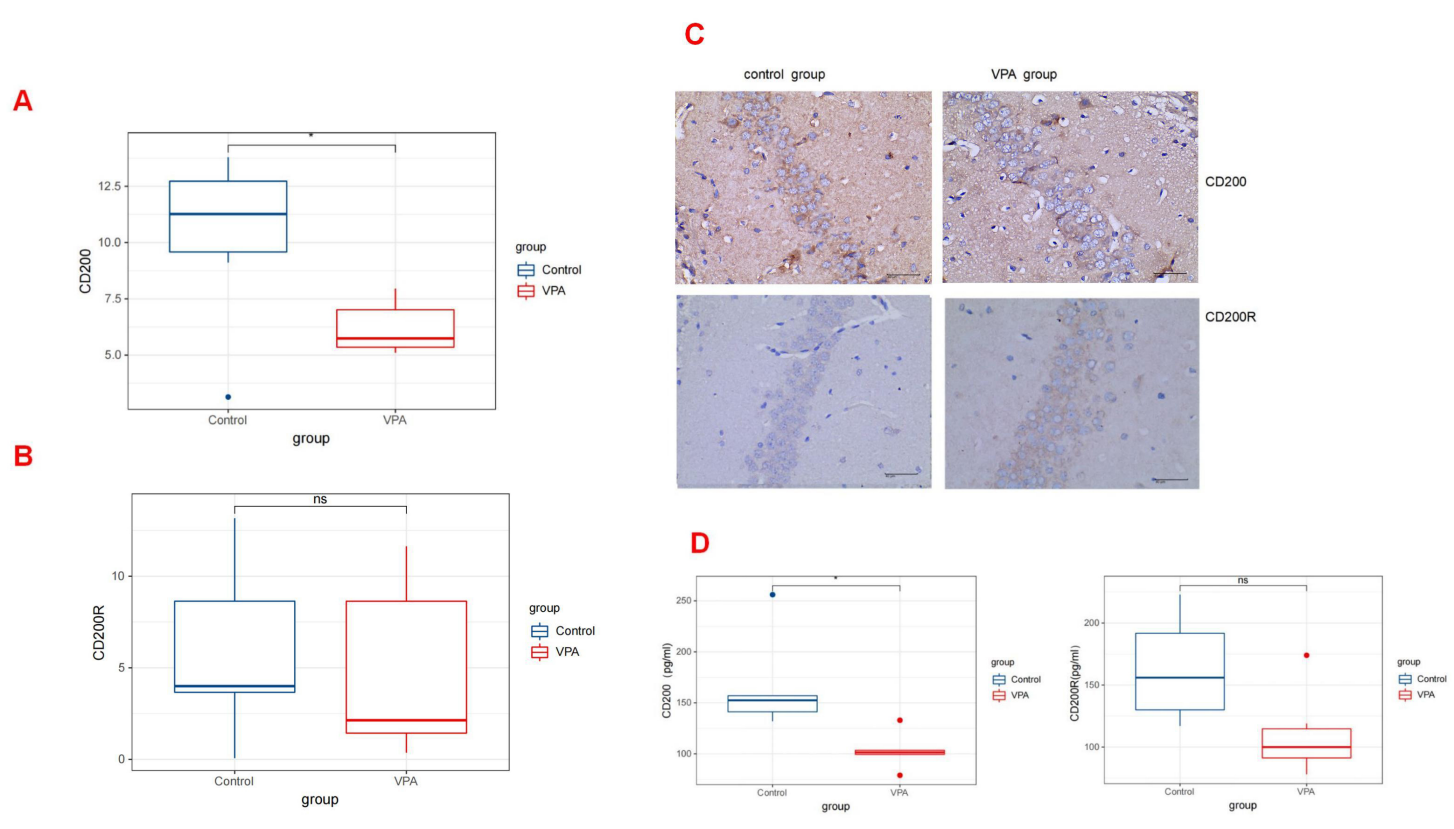
**CD200/CD200R expressions in the Rat Hippocampus**. (A) The expression levels of CD200 by quantitative immunohistochemical analysis in rats treated with valproic acid and saline (*p* < 0.05). (B) Levels of CD200 by ELISA between the VPA group and the control group (*p* < 0.05). (C) Depiction of the corresponding images related to CD200/CD200R expression in the hippocampus from both groups. Scale bar: 40 μm. (D) The expression levels of CD200R by quantitative immunohistochemical analysis and ELISA, separatel (*p* > 0.05). It did not show a statistically significant difference of the level of CD200R between the two groups for VPA-treated rats and controlled rats (*p* > 0.05). However, the quantity of CD200 in rats treated with valproic acid was significantly lower compared with those observed in controlled rats (*p < 0.05). These results showed that exposure to VPA during pregnancy could decrease the level of CD200 of rats offspring. ns, no significance.

By way of the quantitative analysis of immunohistochemistry, the levels of CD200 on postnatal day 30 in rats treated with VPA (median [Q1–Q3]: 6.64 [5.40–7.51]) were significantly lower compared with those observed in rats treated with saline (median [Q1–Q3]: 11.27 [7.62–13.29]; *p* = 0.031) (Fig. 3A). However, the expression levels of CD200R on postnatal day 30 in rats treated with VPA (median [Q1–Q3]: 2.14 [1.11–10.89]) did not exhibit a statistically significant difference compared with those in rats treated with saline (median [Q1–Q3]: 3.99 [2.69–10.92]; *p* =0.441) (Fig. 3B) (Table 1). The ELISA results also indicated a similar distinction between the two groups. A statistically significant difference was observed in CD200 levels between the control and VPA groups (median [Q1–Q3]: 152.50 [137.25, 181.75] vs 101.50 [94.00, 111.25]; *p* = 0.031). However, no significant distinction was found in CD200R levels between the two groups (median [Q1–Q3]: 156.00 [123.00, 205.75] in the control group vs 100.00 [86.25, 132.75] in the VPA group; *p* = 0.139) (Fig. 3D) (Table 2). The corresponding images are shown in Fig. 3C.

### 3.4 No Effect of VPA Treatment on CX3CR1/CX3CL1 Signaling in the Rat Hippocampus

**Fig. 4.**
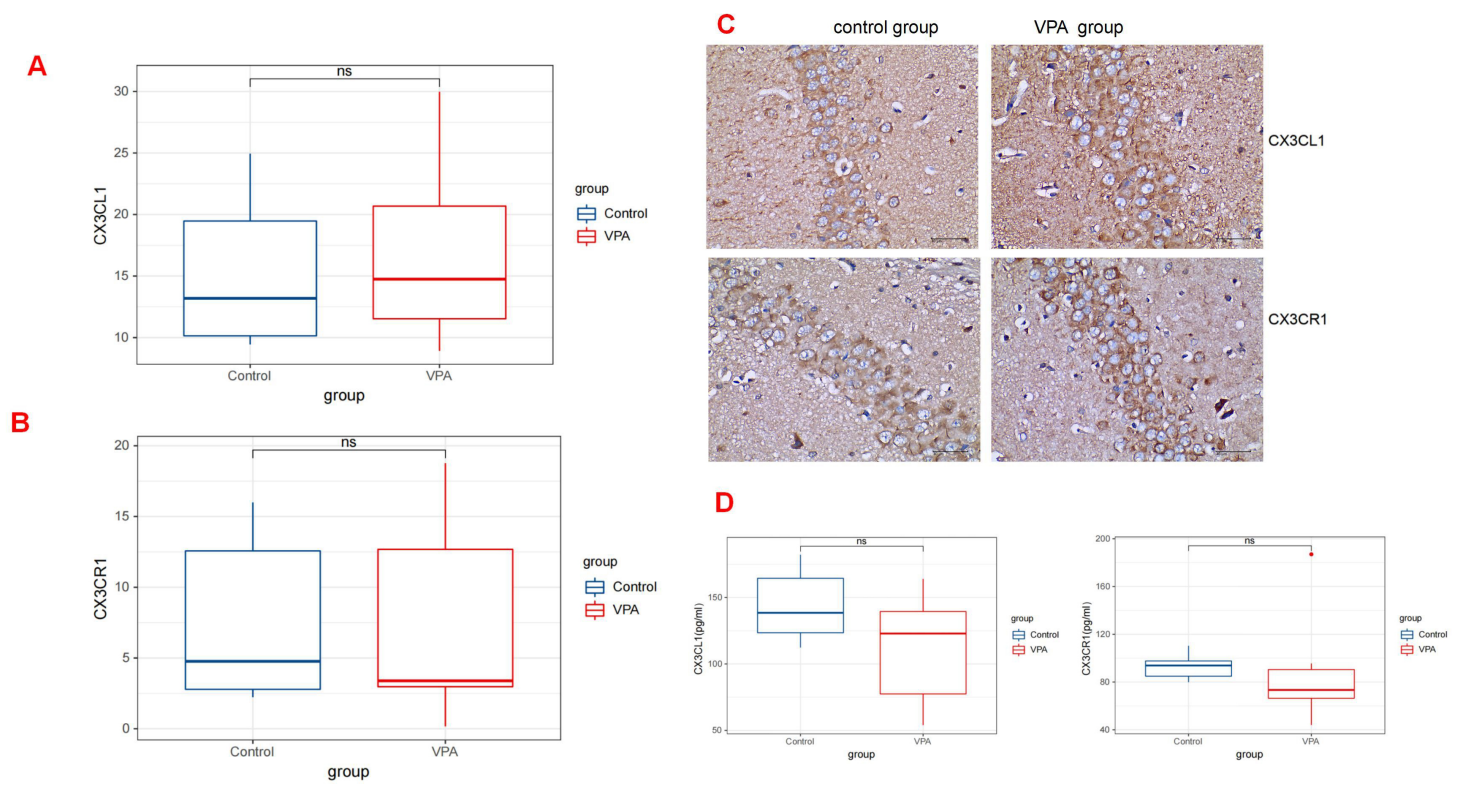
**CX3CR1/CX3CL1 expressions in the Rat Hippocampus**. (A) The expression levels of CX3CL1 by quantitative immunohistochemical analysis in rats treated with valproic acid and saline (*p* > 0.05). (B) Levels of CX3CL1 by ELISA between the VPA group and the control group (*p* > 0.05). (C) Corresponding images related to CX3CR1/CX3CL1 expression in the hippocampus from both groups. Scale bar: 40 μm. (D) The expression levels of CX3CR1 by quantitative immunohistochemical analysis and ELISA, separatel (*p* > 0.05). Rats administered with VPA did not exhibit a statistically significant difference in CX3CR1 expression compared with those in the control group. Similarly, the quantity of CX3CL1 in VPA-treated rats did not demonstrate a significant difference compared with the levels in the control group. It demonstrated that exposure to VPA during pregnancy had no influence on the level of CX3CR1/CX3CL1 of rats offspring. ns, no significance.

By analyzing the immunohistochemistry results, we found that at 30 days post-birth, the rats administered with VPA did not exhibit a statistically significant difference in the level of CX3CR1 expression (median [Q1–Q3]: 4.77 [2.29–15.24]) compared with those given saline (median [Q1–Q3]: 3.39 [2.22–16.47]; *p* = 0.893). Similarly, the quantity of CX3CL1 in VPA-treated rats (median [Q1–Q3]: 14.75 [10.86–23.70]) did not demonstrate a significant difference compared with the levels in saline-treated rats (median [Q1–Q3]: 13.19 [9.67–21.95]; *p* = 0.893) (Fig. 4A,B) (Table 1). The ELISA also indicated no discernible distinction between the two groups. No statistical difference was observed in the CX3CL1 levels between the two groups (median [Q1–Q3]: 138.47 [117.46, 174.84] in the control group vs 122.89 [63.75, 146.20] in the VPA group; *p* = 0.441). Similarly, no significant difference was found in the CX3CR1 levels between the two groups (median [Q1–Q3]: 93.83 [82.31, 100.90] in the control group vs 73.38 [59.46, 118.44] in the VPA group; *p* = 0.139) (Fig. 4D) (Table 2).

Fig. 4A and Fig. 4B show bar graphs summarizing the data analyses on the expression of CX3CR1/CX3CL1 in the hippocampus. The corresponding images are shown in Fig. 4C.

### 3.5 Impact of VPA-Induced Histopathological Changes in the Rat Hippocampus

**Fig. 5.**
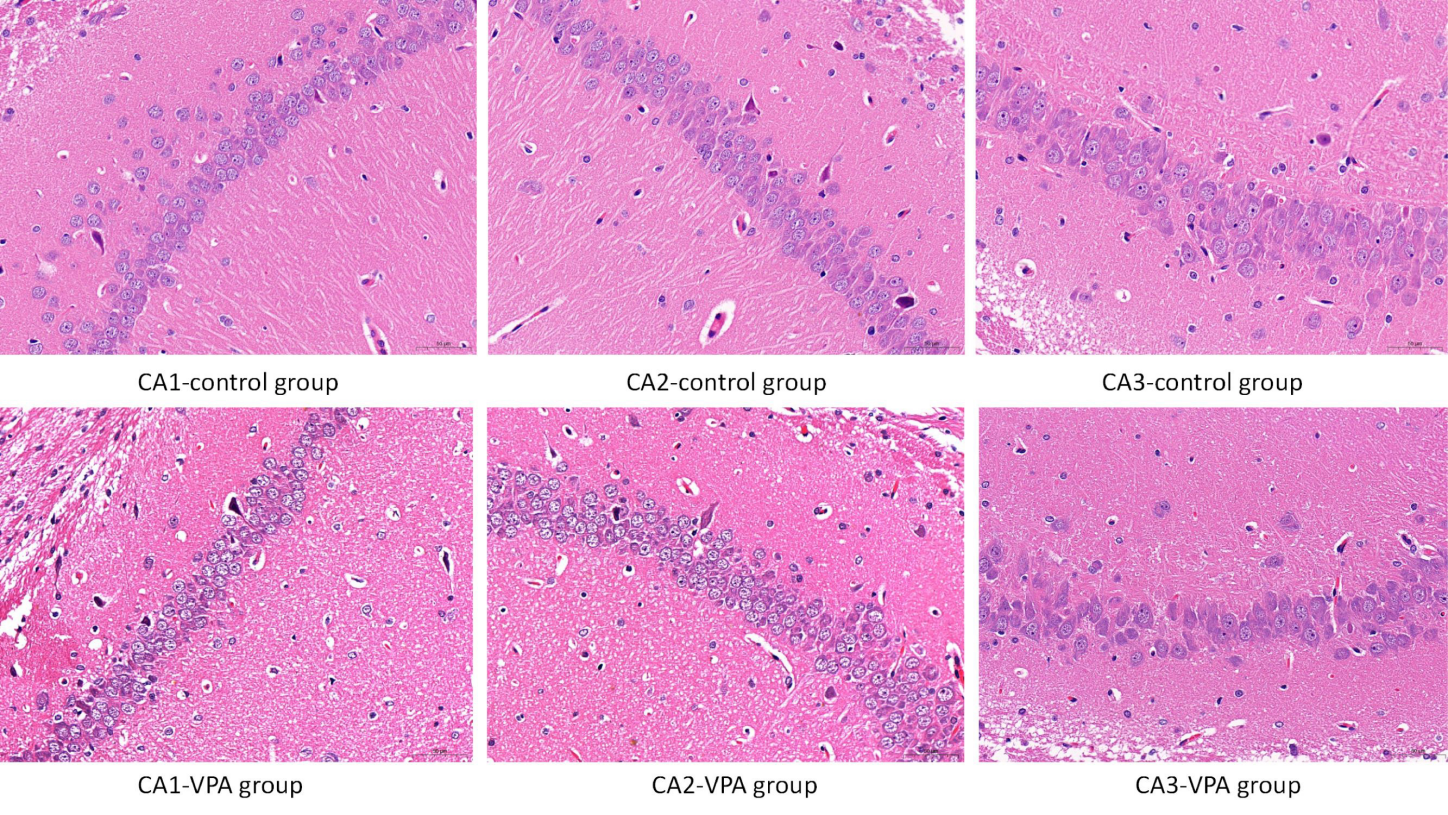
**Neuronal morphological features in the hippocampus assessed using HE staining**. Scale bar: 50μm. No evidence of inflammatory cell infiltration was observed in the CA1, CA2, and CA3 regions of the hippocampus in both control and VPA-treated rats. Furthermore, no pathological alterations were observed in the hippocampus of rats in either group.

The neuronal morphological features in the hippocampus were assessed using HE staining. As shown in Fig. 5, there was no evidence of inflammatory cell infiltration in the CA1, CA2, and CA3 regions of the hippocampus in both control and VPA-treated rats. Additionally, there were no pathological alterations observed in the hippocampus of rats in either group.

## 4. Discussion

As far as we know, this study represents the first effort to examine the influence of VPA exposure during embryonic development on the crosstalk between microglia and neurons through the CD200/CD200R and CX3CL1/CX3CR1 signaling pathways.

This study discovered a decrease in CD200 expression in the hippocampus of offspring rats, while CD200R showed no significant alteration when compared with the control group. This indicates that prenatal exposure to VPA does not notably affect the expression of CD200R in hippocampal microglia but primarily impairs CD200 in hippocampal neurons. This finding suggests that the decreased secretion of CD200 by neurons following VPA treatment has the potential to disrupt the communication between microglia and neurons.

Disruption of the CD200/CD200R system can lead to microglia activation, as CD200/CD200R is crucial for preventing the conversion of microglia into the M2 phenotype. The CD200/CD200R axis plays a crucial role in maintaining homeostasis within the CNS.

The CD200/CD200R system indeed plays an important role in regulating microglial activation and maintaining CNS homeostasis. Disruption of this system can lead to dysregulated microglial activation, which may contribute to neuroinflammation and neurodegenerative diseases [16, 17]. The study by Lyons *et al*. [18] highlights the association between reduced CD200 levels and microglial activation in response to inflammation induced by lipopolysaccharides (LPS). Chamera *et al*. [19] showed that male offspring rats subjected to maternal immune activation exhibit increased vulnerability to microglial deficits triggered by LPS. This susceptibility is attributed to the dysfunction of the CD200-CD200R and CX3CL1-CX3CR1 systems [19]. In another study, it was found that during the early stages of depression, microglia in a specific brain region underwent morphological activation after exposure to chronic social defeat stress. The study revealed that CD200 played a role in inhibiting the excessive microglial activation, thereby decreasing neuroinflammatory responses in the hippocampus [20]. Additionally, another study demonstrated that the loss of CD200 due to neuronal death was a factor that contributed to the activation of microglia following cerebral ischemia [21].

Another consequence of disrupted communication between microglia and neurons could be the manifestation of irregular synaptic function. Xue Jiang *et al*. [22] showed that persistent mild stress exposure resulted in a decrease in the communication molecules (CX3CL1/CX3CR1 and CD200/CD200R) linking neurons and microglia in the hippocampus, which was correlated with synaptic dysfunction. An Alzheimer’s disease model showed that the elevation of neuronal CD200 levels had a specific effect on enhancing cognitive function via the inhibition of microglial activation and secretion, synaptic function enhancement, and prevention of synaptic loss [23]. The study found that by regulating microglial M1/M2 polarization, the activation of the CD200/CD200R1 axis reduced neuroinflammation, synaptic deficits, and cognitive impairment in the hippocampus of aged mice [24].

In the current study, we demonstrated that embryonic exposure to VPA does not influence the microglial cell count during the early postnatal stage of brain development. Moreover, the HE stain analysis revealed no pathological alterations, and there was no observed increase in glial cells within the hippocampus of rats in both experimental groups. Our findings suggest that VPA exposure during embryonic development does not lead to an increase in microglial cells within the rat hippocampus. These results are consistent, to some extent, with previous research findings. The research conducted on VPA-exposed pups showed no significant variations in microglial density among male mice. However, notable alterations in the density of microglial cells were observed between postnatal day 21 and postnatal day 6 in female pups [25].

In a recent study, it was discovered that prenatal exposure to VPA resulted in a notable elevation of IBA1 and mRNA levels of pro-inflammatory cytokines (IL-1β, IL-6, TNF-α) in the cerebral cortex. Nevertheless, this exposure did not impact these indicators in the hippocampus [26]. Several studies have shown varying levels of microglial cell density in offspring following exposure to VPA during pregnancy, and shown that at 5 months of age, female mice exposed prenatally to VPA display an increased total count and density of microglia in the hippocampus [27].

Another study reported that prenatal exposure to VPA in male mice leads to a marked decrease in microglial numbers within the primary motor cortex on both postnatal day 6 and postnatal day 10 [28]. Comparison of these studies indicates that the primary distinction lies in the specific brain regions under investigation and the varying time frames examined. Although sex differences are commonly deemed significant, research involving human subjects has shown that prenatal exposure to VPA may potentially mitigate the conventional male predominance observed in the incidence of ASD [29]. Our research contributes to the growing body of literature on the alteration of microglia levels in the hippocampus of rats 30 days after prenatal exposure to VPA.

Regulating the activation and optimal functioning of microglia is one of the key functions of the CX3CL1/CX3CR1 pathway [14]. Previous studies indicate that the CX3CL1/CX3CR1 pathway can be altered by certain prenatal factors. In mice, an intense immune challenge in late gestation can alter fractalkine signaling by reducing CX3CR1 protein expression in microglial cells [30]. The hippocampus of mice exposed to bacterial LPS exhibited a decrease in CX3CR1 expression levels, a modification associated with behaviors pertinent to maternal immune activation [31]. A deficiency of the chemokine receptor Cx3cr1 in mice leads to a transient decline in microglial cells during early developmental stages, which can result in impaired synaptic pruning mechanisms [32]. However, the results of the current study indicate that there were no significant changes in the levels of CX3CL1 and CX3CR1 within the hippocampus of rats exposed to prenatal VPA, suggesting that this exposure did not significantly impact the expression of these molecules in this brain region.

The mechanism through which prenatal exposure to VPA diminishes CD200 levels during early postnatal development is not fully understood. One potential explanation is that VPA may induce neuroapoptosis and activate a novel calpain-dependent necroptosis pathway involving JNK1 activation and RIP-1 expression, given its neurotoxic effects [33]. Maryam Saadat *et al*. [34] reported that administering VPA during prenatal development resulted in elevated malondialdehyde levels and impaired antioxidant enzyme activity within the hippocampus. Regardless of the specific mechanism, the detrimental and toxic effects of VPA on CD200 expression in neurons may have implications for neuronal synaptogenesis, highlighting the need for additional investigation into the cause and effect relationships.

To summarize, our experiments provide the first evidence of prenatal VPA exposure leading to a reduction in CD200 expression within the hippocampus of mammals, which may have harmful effects on the neuron-microglia axis. The data presented in this study are highly relevant to the pathological conditions associated with ASD. Therefore, further exploration is urgently needed.

Limitations: One limitation of this study is that it focused exclusively on the effects of prenatal exposure to VPA on microglia-neuron intercommunication at a single time point, specifically postnatal day 30. This limited timeframe may not capture the full extent of developmental consequences resulting from prenatal VPA exposure. Long-term follow-up studies are necessary to assess the persistence and potential evolution of the observed alterations in microglia-neuron communication. Additionally, the sample size of each group in this study was relatively small. Lastly, the selection of six offspring from the same pregnant rat for testing might have caused a litter effect. In future research, it will be necessary to increase the sample size to improve statistical power and increase the generalizability of the findings.

## 5. Conclusion

Prenatal VPA exposure could lead to a reduction in CD200 expression within the hippocampus of mammals, which may damage the neuron-microglia axis. The findings suggest that VPA may modify the interactions between microglia and neurons. These disruptions might hurt synaptic connections and then lead to the development of neurodevelopmental disorders, including autism. Further research is needed to elucidate the underlying mechanisms and effects of the pathological condition associated with autism spectrum disorder (ASD).

## Availability of Data and Materials

The datasets used and/or analyzed during the current study are available from the corresponding author on reasonable request.
